# Association between non-high-density lipoprotein cholesterol to high-density lipoprotein cholesterol ratio and cardiovascular disease mortality in patients with type 2 diabetes mellitus and diabetic kidney disease

**DOI:** 10.3389/fendo.2025.1509752

**Published:** 2025-02-28

**Authors:** Zhiyu Li, Hongyang Xu

**Affiliations:** Department of Critical Care Medicine, the Affiliated Wuxi People’s Hospital of Nanjing Medical University, Wuxi People’s Hospital, Nanjing Medical University, Wuxi, China

**Keywords:** non-high-density lipoprotein cholesterol to high-density lipoprotein cholesterol ratio NHHR, diabetic kidney disease, type 2 diabetes mellitus, cardiovascular disease, mortality

## Abstract

**Purpose:**

Non-high-density lipoprotein cholesterol to high-density lipoprotein cholesterol ratio (NHHR) represents an essential lipid index and is closely related to the occurrence and development of diabetes and cardiovascular diseases (CVDs). Therefore, this study is intended to further investigate the association between the NHHR index and the mortality rate of CVDs in patients with type 2 diabetes mellitus (T2DM) and diabetic kidney disease (DKD).

**Methods:**

The research sample was selected from the NHANES (National Health and Nutrition Examination Survey) database, and 5136 individuals were categorized based on quartiles of the NHHR index. Restricted cubic plots and COX regression models were utilized to examine the thresholds and patterns of the NHHR index in relation to the risk of CVDs mortality among T2DM patients as well as those with DKD. Subgroup analyses and p-values were used to evaluate interactions between different variables.

**Results:**

The NHHR index shows a nonlinear association with cardiovascular mortality in two patient groups, following an L-shaped pattern. In individuals with T2DM, a lower NHHR index (<1.68) correlates with an increased risk of death, demonstrating a 72.8% reduction in mortality risk for each unit increase in NHHR below this threshold. Similarly, among patients with DKD, a lower NHHR index (<1.82) is associated with heightened cardiovascular mortality risk, indicating a 48.2% reduction in death risk for each unit increase in NHHR beneath the specified threshold. In patients diagnosed with T2DM, the third quartile of the NHHR index was significantly linked to reduced mortality risk; the association remained consistent even when additional variables were considered [Hazard Ratio (HR), 0.82; 95% Confidence Interval (CI) (0.69-0.97); P=0.019]. Among patients with DKD, cardiovascular mortality was notably higher in the third and fourth quartiles of the NHHR index [Quartile3 HR, 1.57; 95% CI (1.10-2.24), P=0.013; Quartile4 HR, 2.04; 95% CI (1.28-3.26), P=0.003].

**Conclusions:**

The NHHR is below 1.68, and an increase in the NHHR index is associated with a reduced risk of CVD mortality in patients with T2DM. Similarly, when the NHHR falls below 1.82, an elevation in the NHHR index correlates with a decreased risk of CVD mortality in patients with DKD.

## Introduction

Diabetes, particularly T2DM, is often associated with lipid metabolism abnormalities that lead to hyperlipidemia and insulin resistance (1–3). Research indicates that individuals with diabetes have a significantly higher risk of developing CVDs compared to the general population. Furthermore, diabetic patients frequently present with metabolic syndrome features such as hypercholesterolemia and hypertension, which further elevate their risk for cardiovascular events (4). Therefore, it is crucial to investigate the relationship between CVDs risk and abnormal lipid levels in patients with T2DM and its severe complication—DKD.

NHHR is a novel indicator reflecting human lipid metabolism (5). In recent years, the NHHR index has garnered widespread attention for its predictive value regarding the risks of diabetes and CVDs (6). This index integrates information from non-high-density lipoprotein cholesterol (non-HDL-C; total cholesterol minus HDL-C) and HDL-C to comprehensively reflect pro-atherogenic lipid levels within the body (7, 8). Compared to traditional low-density lipoprotein cholesterol (LDL-C), NHHR provides a more accurate depiction of complex lipid metabolism conditions, thereby offering a more comprehensive assessment of cardiovascular risk (9). Studies have suggested that NHHR may serve as an important predictive marker for atherosclerosis and major adverse cardiovascular events (10, 11). The association between NHHR and diabetes primarily manifests in its predictive value concerning all-cause mortality and cardiovascular mortality among individuals with diabetes or prediabetes (12).

It is noteworthy that DKD represents one of the most common microvascular complications while also being a population at elevated risk for CVDs (13). Among these patients, both the incidence rate and mortality due to CVDs are significantly higher than those observed in typical diabetic patients; indeed, CVDs has become their leading cause of death. However, there remains insufficient research on the role of NHHR in predicting cerebrovascular or vascular disease occurrences among patients suffering from advanced complications such as DKD. Consequently, this study aims to utilize data from the NHANES database to further explore the role of NHHR in assessing cardiac risks among individuals diagnosed with T2DM as well as those suffering from DKD.

## Materials and methods

### Research design and subjects

This study included a population sourced from the NHANES database spanning 1999 to 2018. The NHANES is a comprehensive health survey conducted nationwide by the Centers for Disease Control and Prevention (CDC).

**Figure 1:**
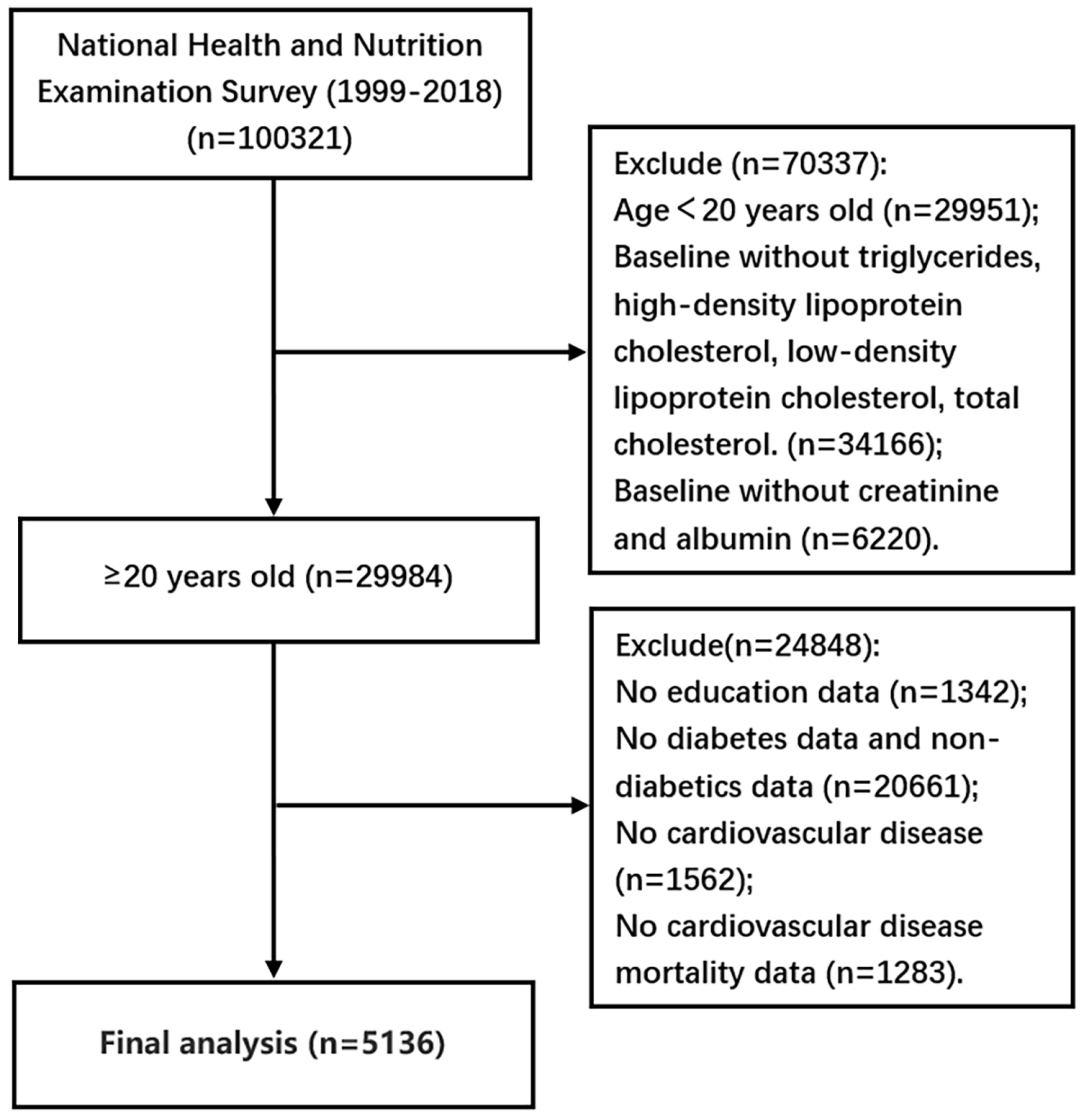
Participants’ screening flow chart.

The inclusion and exclusion criteria for this study were as follows: 1) Inclusion of CVDs, which encompasses coronary heart disease, congestive heart failure, myocardial infarction, stroke, and angina pectoris. The definition of CVD mortality aligns with the relevant guidelines outlined in the International Classification of Diseases, Tenth Revision (ICD-10); 2) Participants with missing data on high-density lipoprotein cholesterol (HDL-C), low-density lipoprotein cholesterol (LDL-C), total cholesterol (TC) and triglycerides (TG) (n=34,166); 3) Exclusion of patients with missing urine creatinine or albumin data (n=6,220); 4) Exclusion of participants under 20 years of age (n=29,951); 5) Exclusion of participants with incomplete cardiovascular data (n=1,562); 6) Exclusion of individuals with missing diabetes status or non-diabetic patients (n=20,661); 7) Deletion of participants with incomplete CVD mortality data (n=1,319); 8) Exclusion of patients with missing educational attainment information (n=1,342). Ultimately, out of 100321 participants screened, we identified 5,136 individuals diagnosed with diabetes and 1,606 cases suffering from DKD (**Figure 1**). Detailed information can be found at https://www.cdc.gov/nchs/nhanes/.

### Survey and laboratory analyses

Plasma biochemicals measured in this study included fasting blood glucose (FBG), HDL-C, LDL-C, TC, TG, serum creatinine and albumin. Variables such as age, sex, education level, smoking status, waist circumference, and body mass index (BMI) were recorded by trained researchers. The categories of race include Mexican American, Other Hispanic, Non-Hispanic White, Non-Hispanic Black, and Other Race. The calculation formula for NHHR index is derived by subtracting HDL-C from TC, followed by dividing the result by HDL-C (14). In this study, the diagnosis of T2DM was established based on a fasting blood glucose value equal to or greater than 7.0 mmol/L. The patient had been diagnosed by a doctor with a CVDs. The diagnosis of DKD in T2DM patients was determined using an all-age spectral correction equation for creatinine to estimate the glomerular filtration rate (eGFR). Specifically, a bilateral eGFR of less than 60 mL/min/1.73 m² (15) and a urinary albumin-to-creatinine ratio (UACR) of ≥30 mg/g were used as criteria for determination.

### Statistical analysis

This study employed sample weighting methods in accordance with NHANES guidelines to mitigate potential biases arising from the complex multi-stage sampling design of NHANES. Categorical variables were weighted by percentage, while continuous variables were adjusted based on their average values and standard deviations. The linear relationship between the NHHR index and mortality from CVDs in T2DM as well as DKD was analyzed using a restricted cubic spline approach, followed by adjustments for multiple confounding factors. Regressions models, including univariate and multivariate Cox regressions, were used to evaluate the NHHR index’s hazard ratios in connection with T2DM and DKD. Model 1 was uncorrected; Model 2 adjusted for sex, age, and BMI, whereas Model 3 included additional factors such asFBG, smoking status, race, TG, TC, hypertension, serum creatinine, albumin, eGFR, waist circumference, education level and UACR. Data analyses were performed using R language (version 4.4.1) and SPSS software (version 27.0), with a significance threshold set at p < 0.05.

## Results

### Characteristics

The study population comprised 5,136 patients with T2DM from the NHANES database. The NHHR index was categorized into four quartiles, revealing significant differences among most variables between groups. Patients in the first quartile had the highest average age of 62.27 years. In the fourth quartile of NHHR, various indicators were generally elevated compared to other groups, including hypertension (21.7%), TG (3.2 mmol/l), LDL-C (3.05 mmol/l), TC (5.92 mmol/l), FBG (157.52 mg/dl), and a higher proportion of individuals with education levels below high school (39.0%). Additionally, urine albumin levels averaged at 140.14 mg/l, while urine creatinine clearance rate (UACR) was recorded at 157.17 mg/g (**Table 1** **).** Among the cohort of 1,606 patients with DKD, further analysis of the fourth quartile revealed that triglyceride levels reached an average of 3.59 mmol/l; this group also exhibited the highest mean age at 69.60 years old. Similarly, within this fourth quartile for NHHR, notable increases were observed across several metrics including TC (5.97 mmol/l), FBG (182.61 mg/dl), and a rise in individuals with education levels below high school to approximately 43.3%. Furthermore, urine albumin levels escalated to an average of 482.31 mg/l while UACR increased significantly to reach values of up to 551.22 mg/g (**Table 2**).

**Table 1:** Weighted baseline characteristics of type 2 diabetes mellitus with cardiovascular disease.

Variables	NHHR				p
	Quartile1 (n=1277)	Quartile2 (n=1290)	Quartile3 (n=1285)	Quartile4 (n=1284)	
Age (years)	62.27 (15.49)	60.51 (14.98)	58.27 (14.63)	54.62 (15.11)	<0.001
BMI (kg/m2)	29.61 (7.53)	31.60 (7.72)	31.97 (7.80)	31.65 (6.85)	<0.001
Sex, n (%)					<0.001
male	636 (49.80)	663 (51.4)	754 (58.7)	859 (66.9)	
female	641 (50.2)	627 (48.6)	531 (41.3)	425 (33.1)	
Hypertension, n (%)					<0.001
yes	228 (17.9)	277 (21.5)	253 (19.7)	279 (21.7)	
no	1049 (82.1)	1013 (78.5)	1032 (81.3)	1005 (78.3)	
TG (mmol/l)	1.05 (0.48)	1.45 (0.67)	1.83 (0.81)	3.24 (3.08)	<0.001
LDL-C (mmol/l)	2.18 (0.70)	2.67 (0.82)	3.06 (0.97)	3.05 (1.69)	<0.001
TC (mmol/l)	4.33 (0.94)	4.70 (0.92)	5.15 (0.94)	5.92 (1.21)	<0.001
HDL-C (mmol/l)	1.65 (0.42)	1.32 (0.26)	1.16 (0.22)	0.97 (0.20)	<0.001
FBG (mg/dl)	140.41 (44.50)	144.16 (47.26)	144.51 (50.78)	157.52 (68.16)	<0.001
Race (%)					<0.001
Mexican American	193 (15.1)	247 (19.1)	295 (23.0)	336 (26.2)	
Other Hispanic	81 (6.3)	103 (8.0)	127 (9.9)	136 (10.6)	
Non-Hispanic White	544 (42.6)	558 (43.3)	523 (40.7)	560 (43.6)	
Non-Hispanic Black	317 (24.8)	268 (20.8)	233 (18.1)	163 (12.7)	
Other Race	142 (11.1)	114 (8.8)	107 (8.3)	89 (6.9)	
Waist circumference (cm)	102.98 (15.95)	107.68 (15.81)	108.83 (15.84)	108.58 (14.51)	<0.001
Smoker, n (%)					0.502
yes	436 (34.1)	407 (31.6)	432 (33.6)	417 (32.5)	
no	841 (65.9)	883 (68.4)	853 (66.4)	867 (67.5)	
Education, n (%)					<0.001
<high school	401 (31.4)	421 (32.6)	441 (34.3)	501 (39.0)	
high school	276 (21.6)	313 (24.3)	307 (23.9)	281 (21.9)	
≥high school	600 (47.0)	556 (43.1)	537 (41.8)	502 (39.1)	
Creatinine (mg/dl)	0.94 (0.46)	0.92 (0.35)	0.94 (0.44)	0.91 (0.31)	0.221
Albumin (mg/L)	74.88 (339.75)	85.27 (353.45)	119.42 (555.51)	140.14 (731.11)	0.005
UACR (mg/g)	75.61 (324.13)	75.89 (311.67)	118.73 (628.84)	157.17 (936.55)	0.001
eGFR, n (%)					<0.001
Stage I	582 (45.6)	603 (46.7)	697 (54.2)	791 (61.6)	
Stage II	493 (38.6)	514 (39.8)	437 (34.0)	373 (29.0)	
Stage III a	130 (10.2)	116 (9.0)	87 (6.8)	70 (5.5)	
Stage III b	53 (4.2)	42 (3.3)	45 (3.5)	39 (3.0)	
Stage IV	15 (1.2)	13 (1.0)	14 (1.1)	11 (0.9)	
Stage V	4 (0.3)	2 (0.2)	5 (0.4)	0 (0.0)	

BMI, Body mass index; FBG, fasting blood glucose; TG, triglycerides; TC, total cholesterol; HDL-C, high-density lipoprotein cholesterol; LDL-C, low-density lipoprotein cholesterol; eGFR, estimated glomerular filtration rate; UACR, urinary albumin-to-creatinine ratio.

**Table 2:** Weighted baseline characteristics of diabetic kidney disease with cardiovascular disease.

Variables	NHHR				P
	Quartile1 (n=402)	Quartile2 (n=395)	Quartile3 (n=403)	Quartile4 (n=406)	
Age (years)	69.60 (11.35)	67.14 (13.83)	63.81 (14.07)	60.29 (15.28)	<0.001
BMI (kg/m2)	29.44 (7.31)	31.40 (7.20)	31.50 (8.68)	32.28 (8.35)	<0.001
Sex, n (%)					<0.001
male	196 (48.8)	183 (46.3)	245 (60.8)	258 (63.5)	
female	206 (51.2)	212 (53.7)	158 (39.2)	148 (36.5)	
Hypertension, n (%)					0.233
yes	63 (15.7)	78 (19.7)	59 (14.6)	71 (17.5)	
no	339 (84.3)	317 (80.3)	344 (85.4)	335 (82.5)	
TG (mmol/l)	1.06 (0.46)	1.59 (0.72)	1.99 (0.88)	3.59 (3.37)	<0.001
LDL-C (mmol/l)	2.03 (0.72)	2.58 (0.91)	2.79 (1.03)	2.77 (1.82)	<0.001
TC (mmol/l)	4.23 (0.97)	4.71 (1.00)	4.98 (0.99)	5.97 (1.28)	<0.001
HDL-C (mmol/l)	1.66 (0.48)	1.33 (0.28)	1.14 (0.23)	0.99 (0.22)	<0.001
FBG (mg/dl)	148.36 (51.95)	154.13 (53.30)	160.79 (63.13)	182.61 (80.15)	<0.001
Race, n (%)					<0.001
Mexican American	52 (12.9)	69 (17.5)	95 (23.6)	109 (26.8)	
Other Hispanic	15 (3.7)	24 (6.1)	29 (7.2)	44 (10.8)	
Non-Hispanic White	189 (47.0)	168 (42.5)	166 (41.2)	168 (41.4)	
Non-Hispanic Black	113 (28.1)	100 (25.3)	82 (20.3)	63 (15.5)	
Other Race	33 (8.2)	34 (8.6)	31 (7.7)	22 (5.4)	
Waist circumference (cm)	104.40 (15.28)	107.92 (14.97)	110.06 (15.69)	111.45 (16.31)	<0.001
Smoker, n (%)					0.247
yes	160 (39.8)	133 (33.7)	158 (39.2)	147 (36.2)	
no	242 (60.2)	262 (66.3)	245 (60.8)	259 (63.8)	
Education, n (%)					0.086
<high school	136 (33.8)	151 (38.2)	153 (38.0)	176 (43.3)	
high school	89 (22.1)	99 (25.1)	98 (24.3)	87 (21.4)	
≥high school	177 (44.0)	145 (36.7)	152 (37.7)	143 (35.2)	
Creatinine (mg/dl)	1.17 (0.71)	1.12 (0.52)	1.15 (0.69)	1.03 (0.46)	0.009
Albumin (mg/L)	200.63 (575.49)	241.61 (580.46)	308.09 (782.69)	482.31 (1375.04)	<0.001
UACR (mg/g)	205.80 (538.45)	226.23 (542.64)	295.03 (808.86)	551.22 (1767.67)	<0.001
eGFR, n (%)					<0.001
Stage I	103 (25.6)	100 (25.3)	149 (37.0)	203 (50.0)	
Stage II	106 (26.4)	119 (30.1)	104 (25.8)	76 (18.7)	
Stage III a	126 (31.3)	114 (28.9)	89 (22.1)	74 (18.2)	
Stage III b	48 (11.9)	47 (11.9)	42 (10.4)	42 (10.3)	
Stage IV	15 (3.7)	13 (3.3)	14 (3.5)	11 (2.7)	
Stage V	4 (1.0)	2 (0.5)	5 (1.2)	0 (0.0)	

BMI, Body mass index; FBG, fasting blood glucose; TG, triglycerides; TC, total cholesterol; HDL-C, high-density lipoprotein cholesterol; LDL-C, low-density lipoprotein cholesterol; eGFR, estimated glomerular filtration rate; UACR, urinary albumin-to-creatinine ratio.

### Association of NHHR index with cardiovascular disease in patients with T2DM and diabetic kidney disease

**Figure 2:**
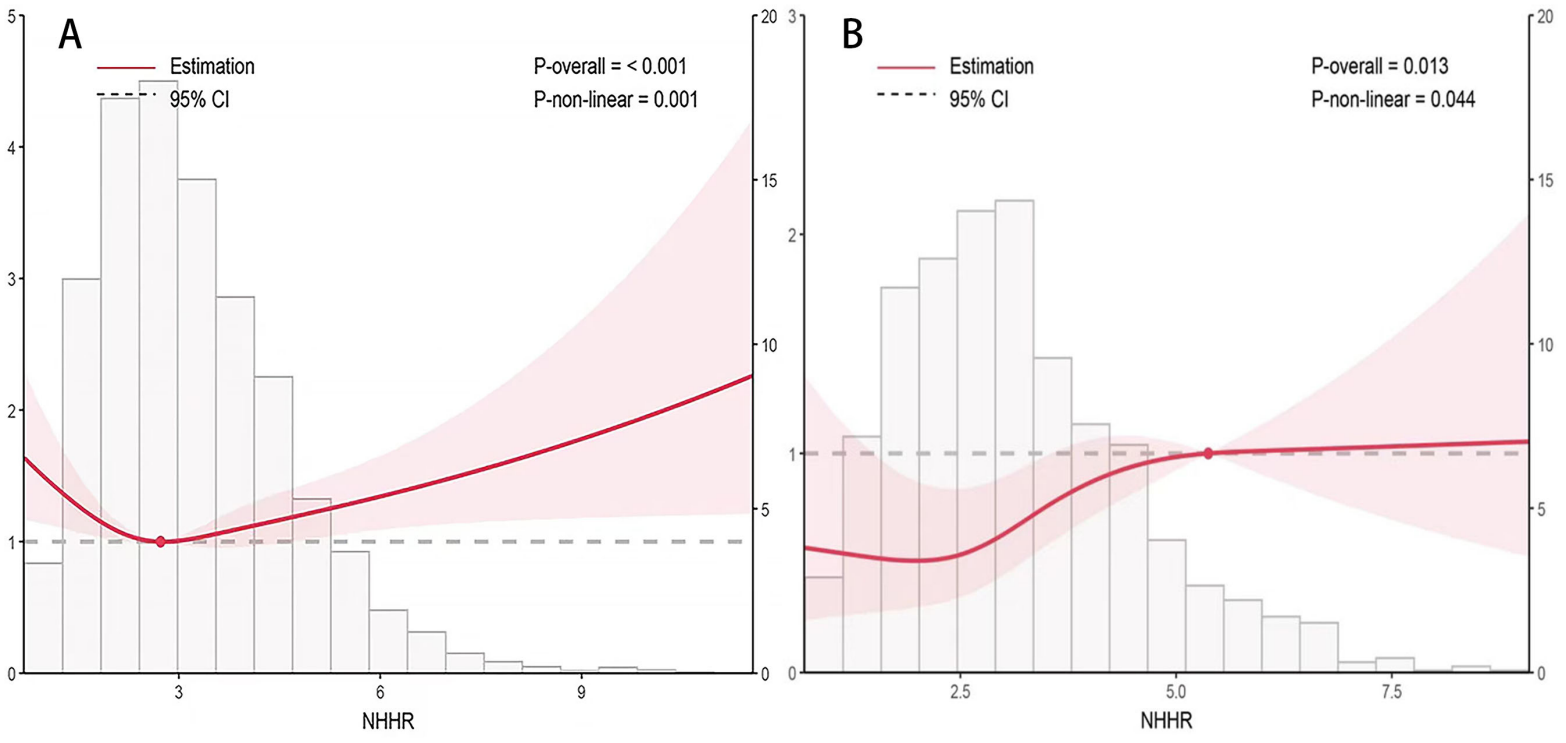
Correlation of the NHHR index with the risk of death from heart disease in patients with type 2 diabetes mellitus **(A)** and diabetic kidney disease **(B)**. Adjusted for age, sex, and BMI, FBG, smoking status, race, TC, TG, hypertension, serum creatinine, albumin, eGFR, waist circumference, education level and UACR. HR, Hazard ratio; CI, Confidence interval.

Overall, the NHHR index exhibited a nonlinear association with CVD mortality risk in both T2DM and DKD patients, characterized by an “L” curve indicating a decreasing risk followed by an increasing risk. The confounding factors adjusted for in **Figure 2** and **Table 3** include age, sex, BMI, FBG, smoking status, race, TC, TG, hypertension, serum creatinine levels, albumin levels, eGFR, waist circumference, education level, and UACR. In T2DM patients, a higher NHHR index was associated with a decreased probability of mortality [HR, 0.925; 95%CI (0.888-0.964), P<0.001]. Two-segmented linear regression analysis identified the inflection point of the NHHR index at 1.68. Below the specified threshold, an increase in the NHHR index was linked to a 72.8% decrease in the risk of death [HR, 0.372; 95%CI (0.254-0.547), P<0.001]. On the other hand, for values above 1.68, the NHHR index did not demonstrate a substantial association with the likelihood of death [HR, 0.959; 95%CI (0.919-1.001), P=0.054] (**Figure 2A**, **Table 3**).

**Table 3:** Threshold effect analysis of the NHHR index and cardiovascular disease mortality in type 2 diabetes mellitus and diabetic kidney disease.

Outcomes	HR, 95%CI, P value
Diabetes mortality	
Model 1 Fitting model by standard linear regression	0.925(0.888-0.964)0.001
Model 2 Fitting model by two-piecewise linear regression	
Inflection point	1.68
<1.68	0.372(0.254-0.547)0.001
≥1.68	0.959(0.919-1.001)0.054
P for likelihood ratio test	<0.001
Diabetic kidney disease mortality	
Model 1 Fitting model by standard linear regression	0.921(0.872-0.972)0.003
Model 2 Fitting model by two-piecewise linear regression	
Inflection point	1.82
<1.82	0.502(0.347-0.727)0.001
≥1.82	0.961(0.907-1.02)0.19
P for likelihood ratio test	0.002

HR, Hazard ratioz; CI, Confidence interval; adjusted for age, sex, and body mass index (BMI), fasting blood glucose (FBG), smoking status, race, triglycerides (TG), total cholesterol (TC), hypertension, serum creatinine, albumin, estimated glomerular filtration rate (eGFR), waist circumference, education level and urinary albumin-to-creatinine ratio (UACR).

A similar nonlinear relationship was noted among DKD patients as well; here too, a higher NHHR index corresponded to reduced probability of cardiovascular death [HR, 0.921; 95%CI(0.872-0.972), P<0.001], with an inflection point established at 1.82—higher than that found in T2DM patients’ cohort. When the NHHR index fell below this value of 1.82, there was a notable decrease of approximately 48.2% per unit increase in probability of death [HR, 0.502;95%CI (0.347-0.727), P<0.001]; however, when it exceeded this threshold, no significant correlation emerged between the NHHR index and probability of death [HR, 0.0961;95%CI (0.0907-1.02), P=0.19] (**Figure 2B**, **Table 3**).

### Univariate and multivariate COX regression models

In univariate COX regression analyses, the NHHR index in the fourth quartiles demonstrated statistically significant correlations with cardiovascular mortality risk among diabetic patients. It is noteworthy that Model 2 adjusted for several confounding variables, including age, sex, and BMI, revealing a strong association. Furthermore, in Model 3, which accounted for additional variables such as FBG, smoking status, race, TC, TG, hypertension, serum creatinine levels, albumin levels, eGFR, waist circumference, education level and UACR alongside age and sex and BMI; the NHHR index demonstrated a more pronounced relationship with the mortality risk among patients with T2DM [HR: 0.82; 95% CI (0.69-0.97), P=0.019] (**Table 4**). In Model 3, a correlation was observed between the risk ratio of DKD leading to death and the third quartile of the NHHR index [HR: 1.51; 95%CI (1.06-2.14); P = 0.022]. In addition, the fourth quartile of the NHHR index and the DKD mortality risk ratio were also significant in Model 3 [HR, 2.04; 95% CI (1.28-3.26); P = 0.003] (**Table 4**).

**Table 4:** Univariate and multivariate COX regression modeling of the NHHR index and cardiovascular disease in type 2 diabetes mellitus and diabetic kidney disease.

NHHR	Model 1	P	Model 2	P	Model 3	P
	HR (95%CI)		HR (95%CI)		HR (95%CI)	
Diabetes mortality						
Quartile1	1.00 (Reference)		1.00 (Reference)		1.00 (Reference)	
Quartile2	0.88 (0.75 ~ 1.03)	0.115	0.89 (0.76 ~ 1.05)	0.161	0.88 (0.75 ~ 1.04)	0.14
Quartile3	0.75 (0.64 ~ 0.88)	<.001	0.80 (0.68 ~ 0.95)	0.011	0.82 (0.69 ~ 0.97)	0.019
Quartile4	0.75 (0.64 ~ 0.88)	<.001	0.94 (0.77 ~ 1.13)	0.498	0.95 (0.78 ~ 1.15)	0.585
Diabetic kidney disease mortality						
Quartile1	1.00 (Reference)		1.00 (Reference)		1.00 (Reference)	
Quartile2	0.89 (0.72 ~ 1.10)	0.291	0.96 (0.77 ~ 1.19)	0.707	1.34 (1.01 ~ 1.78)	0.044
Quartile3	0.76 (0.61 ~ 0.94)	0.014	0.76 (0.61 ~ 0.94)	0.013	1.51 (1.06 ~ 2.14)	0.022
Quartile4	0.77 (0.62 ~ 0.96)	0.021	0.79 (0.63 ~ 0.98)	0.033	2.04 (1.28 ~ 3.26)	0.003

HR, Hazard ratio; CI, Confidence interval. Model 1: unadjusted; Model 2: corrected for age, sex, and body mass index (BMI); Model 3: Model 2+, fasting blood glucose (FBG), smoking status, race, triglycerides (TG), total cholesterol (TC), hypertension, serum creatinine, albumin, estimated glomerular filtration rate (eGFR), waist circumference, education level and urinary albumin-to-creatinine ratio (UACR).

### Analysis subgroups

**Figure 3:**
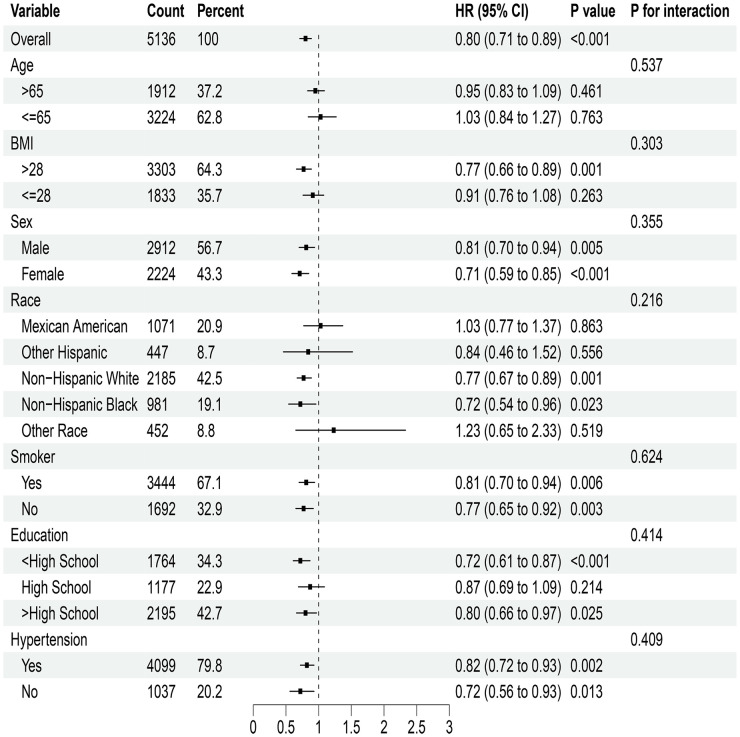
Stratified analysis of the NHHR index and mortality from cardiovascular disease in type 2 diabetes mellitus. HR, Hazard ratio; CI, Confidence interval.

**Figure 4:**
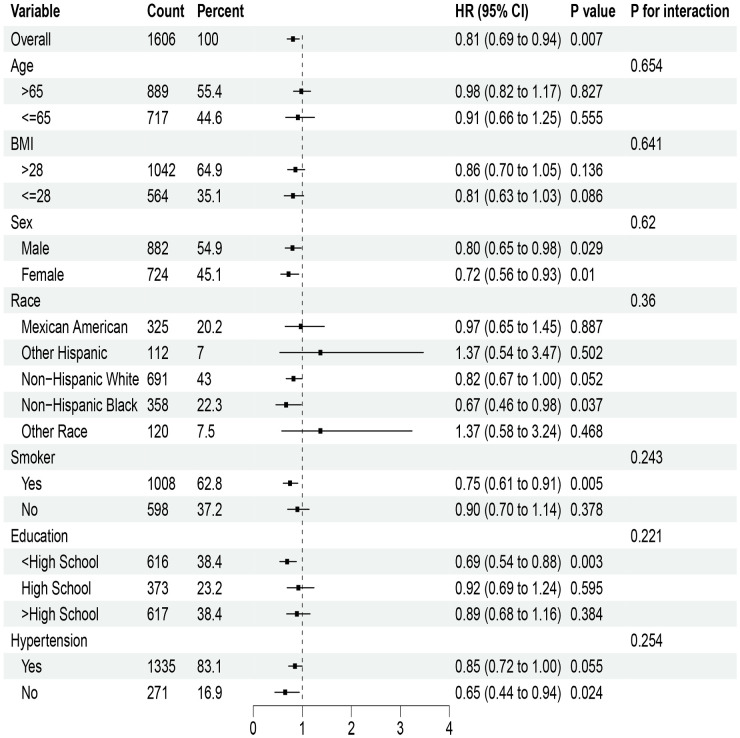
Stratified analysis of the NHHR index and mortality from cardiovascular disease in diabetic kidney disease. HR, Hazard ratio; CI, Confidence interval.

In the analysis of NHHR index and cardiovascular mortality risk ratio for T2DM, notable correlations were observed with elevated BMI, sex, tobacco use, education level, and hypertension (**Figure 3**). However, no meaningful interactions were observed in these subgroups. For DKD mortality risk ratios related to the NHHR index, significant findings emerged among different genders, smokers, individuals with lower education levels, and non-hypertensive patients; yet their interactions showed no statistical significance (**Figure 4**).

## Discussion

This study confirms a significant “L” shaped relationship between the NHHR index and the risk of cardiovascular mortality in patients with T2DM and DKD. Notably, our findings indicate that when the NHHR index falls below specific thresholds (1.68 for T2DM and 1.82 for DKD), an increase of one unit in the index is associated with a marked reduction in mortality risk. This suggests that within a certain range, elevated NHHR indices may be linked to lower mortality risks.

This study reveals a consistent association between the NHHR index and patients with T2DM, aligning with previous research findings (12, 16, 17). These studies also indicate that dyslipidemia is a significant predictor of adverse outcomes in T2DM and DKD patients. However, when the NHHR index exceeds a specific threshold, this association becomes non-significant. This non-linear relationship suggests that the NHHR index may not merely serve as a straightforward risk prediction indicator; rather, its underlying mechanisms could be more complex—potentially related to different stages of disease progression or individual patient variations. Furthermore, it is noteworthy that in DKD patients, this inflection point is higher than in T2DM patients, which may reflect differences in disease severity and prognostic risk between these two groups.

The NHHR could be an indicator of the progression of atherosclerosis (6), which creates a prerequisite for the occurrence of CVDs in T2DM (11, 18, 19). Elevated levels of non-HDL-C, including LDL-C and very low-density lipoprotein cholesterol (VLDL-C), promote LDL oxidation, leading to the formation of foam cells and plaque buildup in the arterial wall, thus initiating atherosclerosis. On the other hand, HDL-C has anti-atherogenic properties such as reverse cholesterol transport, playing a crucial role in reducing the incidence of CVDs (20, 21). A study in China has shown that the NHHR index provides a more comprehensive assessment than LDL-C or HDL-C alone (22). Additionally, it has been found that the NHHR can be used as an important marker for early detection of high-risk carotid plaques (23). An elevated NHHR is often linked to insulin resistance. Excessive non-HDL lipoproteins disrupt insulin signaling pathways and reduce insulin sensitivity, ultimately leading to higher blood glucose levels (24, 25). Furthermore, components within non-HDL lipoproteins and their oxidation products can trigger chronic low-grade inflammation. The inflammatory reaction has a negative effect on the functioning of pancreatic β-cells, leading to a decrease in insulin secretion and worsening the progression of T2DM (26, 27).

DKD is a severe complication of T2DM (28), leading to potential renal failure and an increased susceptibility to CVDs (29). The progression of DKD can be worsened by hyperlipidemia, which also raises the risk of mortality (30, 31). Additionally, kidney disease itself may impact lipid metabolism, resulting in an elevated NHHR (32). This increase in ratio could be linked to various molecular mechanisms, including hyperlipidemia-induced inflammatory responses in vascular endothelial cells and low HDL-C levels promoting atherosclerosis development in coronary arteries (33–35). Furthermore, research has shown that the NHHR index is a reliable predictor for coronary slow flow (CSF) (36, 37).

### Study strengths and limitation

The research discovered a correlation that is not linear between the NHHR index and individuals suffering from both T2DM and DKD with CVDs, offering potential insights into the underlying pathological mechanisms of these conditions. A higher NHHR index is linked to a reduced mortality risk, indicating its potential as a valuable predictive marker. Furthermore, the study also identified a turning point in the NHHR index for patients with T2DM and DKD, which may help us determine the future treatment targets for these diseases.

While this study used a relatively large sample size, there is still a chance of selection bias impacting the generalizability of its findings. The study focused on the trends that existed between the NHHR index and the risk of death and did not explore other potential predictors. Additionally, because this study had a retrospective design, there is an inherent risk of bias influencing its results. It is possible that researchers did not adequately consider confounding factors affecting mortality risks in T2DM and DKD, which could lead to biased outcomes.

### Conclusions

The NHHR index exhibits a non-linear relationship with the risk of CVD mortality in patients with DKD and T2DM. Specifically, when the NHHR is below 1.68, an increase in the NHHR index is associated with a reduction in CVD mortality risk among individuals with T2DM. Similarly, when the NHHR falls below 1.82, an elevation in the NHHR index correlates with a decreased risk of CVD mortality in patients suffering from DKD. These findings may hold potential value for assessing prognosis using the NHHR index within these two populations; however, further prospective studies are necessary to validate these results.

## Data Availability

Publicly available datasets were analyzed in this study. This data can be found here: the datasets generated and analyzed during the current study are available in the NHANES repository (https://www.cdc.gov/nchs/nhanes/index.htm).
